# The Relation Between Parenting Stress and Children’s Social Anxiety in Chinese Family: The Roles of Maladaptive Parenting and FKBP5 Gene Variation

**DOI:** 10.3390/bs16061015

**Published:** 2026-06-17

**Authors:** Beibei Zhang, Deqiang Wang, Huijuan Di, Yue Li, Shizhu Gou, Yaqi Sun, Xue Gong, Tiantian Bi

**Affiliations:** 1 School of Teacher Education, Hebei Normal University, Shijiazhuang 050024, China; zhangbei1992@mail.hebtu.edu.cn (B.Z.); dihuijuan100@hebtu.edu.cn (H.D.); goushizhu@stu.hebtu.edu.cn (S.G.); syq5280@stu.hebtu.edu.cn (Y.S.); 2 School of Home Economics, Hebei Normal University, Shijiazhuang 050024, China; wangdeqiang@hebtu.edu.cn; 3 Department of Preschool-Education, Shijiazhuang Preschool Teachers College, Shijiazhuang 050228, China; 15081856664@163.com; 4 Department of Psychology, Normal College, Qingdao University, Qingdao 266071, China; gongxuedw@163.com

**Keywords:** parenting stress, social anxiety, maladaptive parenting, FKBP5 gene, Chinese family

## Abstract

Background: Parenting stress is a known risk factor for children’s social anxiety, yet the mediating and moderating mechanisms underlying this relationship remain underexplored, particularly regarding gene–environment interactions. This quantitative, cross-sectional study, grounded in diathesis-stress and family process theories, examined whether maladaptive parenting mediates the link between parenting stress and children’s social anxiety, and whether FKBP5 gene variation moderates this mediation. Methods: A sample of 1774 fourth- to sixth-grade students (aged 10–14 years) and their parents participated. Parenting stress and maladaptive parenting were parent-reported, children’s social anxiety was self-reported, and children’s FKBP5-related cumulative genetic score was derived from four SNPs (rs4713916, rs1360780, rs3800373, rs9296158). Moderated mediation analyses were conducted. Results: Parenting stress was significantly and positively associated with children’s social anxiety. Maladaptive parenting partially mediated this relationship. The FKBP5 showed a marginally significant moderating effect, with simple slope analysis suggesting parenting stress was more strongly associated with child social anxiety among children with higher genetic risk. No moderating effect was found for the path from maladaptive parenting to social anxiety. Conclusions: Parenting stress is associated with children’s social anxiety both directly and indirectly through maladaptive parenting, with FKBP5-related cumulative genetic risk potentially moderating the direct effect. These findings offer preliminary evidence that may inform preventive interventions targeting parenting stress, although replication is needed.

## 1. Introduction

Mental health problems in children are becoming increasingly prominent and exhibit a global trend of rising prevalence. Social anxiety in children, as a common mental health concern, is characterized by persistent fear of social situations, avoidance, and negative self-evaluation (Yuan, 2025). A meta-analysis estimates that approximately 4.7% of children and 8.3% of adolescents meet the diagnostic criteria for social anxiety disorder globally, with prevalence increasing progressively across developmental stages (Salari et al., 2024). This condition exacts a heavy immediate toll, significantly disrupting academic performance, school attendance, and peer relationships (Halidu & Kotera, 2024), while also increasing vulnerability to suicidal ideation (Leigh et al., 2023). The long-term consequences are equally alarming; longitudinal evidence shows that persistent childhood social anxiety serves as a robust predictor of major depressive disorder, generalized anxiety disorder, panic disorder, agoraphobia, and obsessive–compulsive disorder in young adulthood (Krygsman & Vaillancourt, 2022). Therefore, it is imperative to identify the key factors and mechanisms underlying the development of social anxiety in children.

Within the family context, parents, as primary caregivers, are subject to considerable parenting stress. Distinct from general life stress, parenting stress not only adversely affects parents’ own mental health but also exerts a more specific influence on the emergence of children’s social anxiety through its impact on daily parent–child interactions. However, parenting stress may operate indirectly by altering parenting behaviors, and this process may vary depending on children’s genetic predispositions. Although numerous studies have focused on G × E (gene–environment) interactions in the development of children’s social anxiety, few have elucidated precisely how genetic factors and the family environment interact. Accordingly, the present study, drawing on a sample of 1774 fourth- to sixth-grade students and their parents, aims to examine whether parental parenting stress is “transmitted” to children through parenting behaviors (particularly maladaptive parenting behaviors), thereby contributing to children’s social anxiety, and to determine the moderating role of children’s FKBP5 (FK506 binding protein 5) gene in this transmission pathway.

### 1.1. The Relationship Between Parenting Stress and Children’s Social Anxiety

Parental parenting stress refers to the specific stress experienced by parents in fulfilling the parenting role, with sources including difficulties in managing children’s behavior, parent–child conflict, insufficient self-efficacy, and a lack of external support (Luo et al., 2019). Generally, parents may experience varying degrees of parenting stress across different stages of child-rearing, and this stress is often particularly pronounced when children are of school age (Anthony et al., 2005).

From a theoretical perspective, the family environment constitutes one of the most immediate and enduring proximal influences on children’s social anxiety. According to the developmental psychopathology perspective on parenting stress (Crnic, 2024), high levels of parenting stress deplete parents’ emotional regulatory resources and diminish their sensitivity and responsiveness to children’s needs. Through mechanisms such as emotional contagion, insecure attachment, and social learning, children may gradually acquire anxious, avoidant, and withdrawn response patterns.

Empirical research has documented a positive association between parenting stress and children’s social anxiety. For example, Çelebi and Acar (2024) found that parenting stress was positively associated with child anxiety in a sample of Turkish mothers of preschoolers. In China, a study by Y. Liu and Jin (2025) reported that parenting stress was generally high and positively correlated with anxiety in children aged 3–6 years. In China, rapid social change and educational pressure have been linked to elevated parenting stress (Luo et al., 2019). Studies have also extended these findings to school-aged samples: Goldsmith et al. (2022), following 868 twin families (aged 8–15), found that parenting stress significantly strengthened the predictive effect of behavioral inhibition on social anxiety.

Notably, although several studies have included school-aged children and adolescents (e.g., Goldsmith et al., 2022), most research has focused on preschoolers, and relatively few have separately examined upper elementary school students (grades 4–6). This developmental period is characterized by increased social comparison and peer interaction, while children remain highly dependent on the family environment. Thus, further exploration of the mechanisms linking parenting stress to social anxiety specifically in this age group is warranted. The present study aims to address this gap.

### 1.2. The Mediating Role of Maladaptive Parenting

Maladaptive parenting encompasses various forms, including harsh and psychological control. Harsh punishment refers to parents’ use of corporal punishment or aggressive behaviors to regulate children’s conduct (Cuartas et al., 2025); psychological control denotes parents’ manipulation of children’s inner world through guilt induction, withdrawal of affection, or invalidation of personal feelings (Choe et al., 2023). These two dimensions—coercive behavioral control and emotional manipulation—together constitute a significant risk pathway for the development of social anxiety in children (Qiu, 2024; Thompson et al., 2024).

Family Interaction Theory (Moed, 2024) provides a theoretical framework for understanding how parental parenting stress escalates into maladaptive parenting toward children. In high-stress family environments, a “coercive cycle” tends to emerge between parents and children: heightened parenting stress elicits negative controlling behaviors from parents, which in turn exacerbate children’s emotional dysregulation, trapping family interactions in a destructive spiral. Empirical evidence supports this pathway. For instance, Yoon et al. (2023) found a significant positive association between parenting stress and child maltreatment risk among Asian immigrant parents. Han et al. (2024) conducted a longitudinal study showing that parenting stress at earlier time points was significantly associated with later maladaptive parenting.

Conversely, maladaptive parenting has been shown to predict children‘s social anxiety. Rowe et al. (2015) found that coercive parenting predicted increases in adolescents’ social anxiety symptoms. A meta-analysis by J. Liu et al. (2023) confirmed a significant positive association between maladaptive parenting and social anxiety, with a stronger effect for emotional abuse.

Integrating these two lines of evidence, it is plausible that maladaptive parenting mediates the relationship between parenting stress and children‘s social anxiety. Importantly, genetic factors may further moderate this mediation, as discussed below. Notably, children’s genetic background may moderate the effects of these environmental risks. Among relevant genes, the FKBP5 gene encodes a protein involved in regulating the HPA axis stress response and is a key candidate for studying gene-environment interactions.

### 1.3. The Moderating Role of the FKBP5 Gene

The FKBP5 gene (FK506 binding protein 5) encodes the FKBP51 protein, which acts as a negative regulator of the glucocorticoid receptor. When an individual faces stress, FKBP51 reduces receptor affinity, thereby affecting cortisol levels and the magnitude of the stress response (Zannas & Binder, 2014). Thus, FKBP5 polymorphisms are closely linked to HPA axis function and stress-related disorders.

According to the diathesis–stress model posits that individuals carrying “risk” alleles are more susceptible to the adverse effects of negative environments, thereby manifesting more problem behaviors (Grillo, 2025). Specifically, children exposed to high-stress or negative parenting environments show repeated activation of their stress response systems. For children carrying FKBP5 risk alleles, impaired glucocorticoid receptor negative feedback may lead to chronically elevated cortisol levels, which in turn may compromise prefrontal cortex function and emotion regulation (Zhou & Gong, 2023). Consequently, these children may have difficulty disengaging from threat-related cues and may be more prone to anxiety (Xing et al., 2025).

The four FKBP5 SNPs selected for this study—rs4713916, rs1360780, rs3800373, and rs9296158—have been widely investigated in prior gene–environment interaction research. A comprehensive meta-analysis by Fan et al. (2021) reported that rs1360780 was associated with depression risk, and that rs4713916 and rs3800373 were also implicated in depression susceptibility. Furthermore, Isaksson et al. (2016) found that a risk haplogenotype including the minor alleles of rs3800373, rs9296158, rs1360780, and rs4713916 was associated with higher anxiety ratings among females exposed to violence. These four SNPs have also been used together in previous studies examining the moderating role of FKBP5 in the link between childhood adversity and mental health outcomes (e.g., Zhou & Gong, 2023; Zeng et al., 2024). Therefore, constructing a cumulative genetic score based on these four loci provides a theoretically and empirically grounded index of FKBP5-related genetic sensitivity.

Prior research on gene-family environment interactions in children‘s social anxiety has several limitations. First, most studies have been confined to single-locus analyses, which suffer from small effect sizes and poor replicability. Second, existing research has typically tested the overall “family adversity → child social anxiety” interaction without distinguishing between different pathways within a mediation model (i.e., whether genes moderate the direct path or the indirect path). These pathways have different theoretical and intervention implications.

### 1.4. Theoretical Framework

The present study is guided by an integrated framework combining three theoretical perspectives: the bioecological model, family process theory, and the diathesis-stress model. The bioecological model (Bronfenbrenner & Morris, 2006) emphasizes that child development is driven by proximal processes, with daily parent–child interactions being the most important proximal environment. Extensive research has confirmed that parental psychological states (e.g., parenting stress) affect children‘s emotional and behavioral problems through these interactions (Crnic, 2024). Family process theory (Moed, 2024) further proposes that high parenting stress triggers a “coercive cycle”—parents resort to harsh punishment or psychological control, which exacerbates child dysregulation. The diathesis-stress model (Grillo, 2025) holds that individuals carrying risk alleles are more sensitive to negative environments.

Despite empirical support for each theory, two important gaps exist. First, although the bioecological model emphasizes mediation through proximal processes, most studies have tested only the direct association between parenting stress and child outcomes or separately examined maladaptive parenting as a mediator, without integrating all three into a single mediation model. Second, the diathesis-stress model has been applied to child social anxiety mainly in single-locus analyses that test only the overall environment × gene interaction, without distinguishing which path within a mediation model is moderated—the direct path (parenting stress → social anxiety) or the second-stage path (maladaptive parenting → social anxiety). Different paths have different intervention implications.

Based on the above, we propose an integrated framework: parenting stress affects children’s social anxiety through the mediation of maladaptive parenting; the FKBP5 cumulative genetic score, as an index of biological sensitivity, may moderate the direct path and/or the second-stage path.

### 1.5. The Present Study: Research Questions and Hypotheses

To address the above-mentioned gaps, the present study draws on a sample of 1774 fourth- to sixth-grade students (aged 10–14 years) and their parents to examine the proposed moderated mediation model. Specifically, we test whether maladaptive parenting mediates the association between parenting stress and children’s social anxiety, and whether FKBP5 gene variation moderates this mediation.

**Figure 1:**
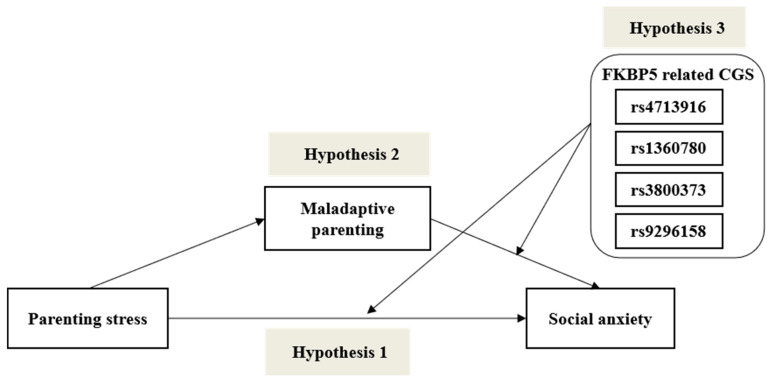
Conceptual framework of the proposed moderated mediation model.

Based on the theoretical framework and literature review, this study addresses the following research questions (see Figure 1):

Research Question 1: Is parenting stress directly associated with children‘s social anxiety?

Research Question 2: Does maladaptive parenting mediate the association between parenting stress and children’s social anxiety?

Research Question 3: Does the FKBP5-related cumulative genetic score moderate the mediation model? If so, does it moderate the direct path (parenting stress → social anxiety) or the second-stage path (maladaptive parenting → social anxiety), or both?

Accordingly, we propose the following hypotheses:

**Hypothesis** **1.** *Parenting stress is significantly and positively associated with children‘s social anxiety.*

**Hypothesis** **2.** *Maladaptive parenting partially mediates the association between parenting stress and children’s social anxiety.*

**Hypothesis** **3.** *The FKBP5 cumulative genetic score moderates the mediation model. Given the exploratory nature of the genetic moderation, we do not hypothesize a priori whether moderation occurs on the direct path, the second-stage path, or both.*

## 2. Materials and Methods

### 2.1. Participants

The data utilized in this study were gathered from two randomly selected elementary schools in a city (Linyi, Shandong Province) in Northeast China. Participation was extended to all fourth- to sixth-grade students and their parents. The positive cooperation of the school administrations led to an approximate participation rate of 98% across both institutions. Official school records indicated that the majority of the students came from middle-class families, and most participating parents had attained at least a middle school education. In total, the study included 1774 Han Chinese adolescents aged between 10 and 14 years old (42.5% girls; *M*_age_ = 11.38 years, *SD* = 0.93).

### 2.2. Procedure

This study received ethical approval from the Human Research Ethics Committees of Hebei Normal University. Informed consent was obtained from all participating students and from the primary caregiver (either the father or the mother) per family prior to data collection. Participation was entirely voluntary, and participants were informed that they could withdraw at any time without any negative consequences. All data were anonymized to protect participant confidentiality. No physical or psychological harm was anticipated or reported during the study. As a token of appreciation, participants received small gifts upon completion of the questionnaires. Participants were explicitly assured that all collected data would be treated with strict confidentiality, and access to completed questionnaires would be restricted exclusively to the research team for analytical purposes.

Social anxiety data were collected from students during classroom sessions. Before the survey administration, trained research assistants provided uniform verbal and written instructions to students in each class, allowing them sufficient time to complete the assessments at their own pace. Parenting stress and maladaptive parenting were reported by one parent per family via parental questionnaires, which was distributed to students to take home and return to researchers the following day after completion. Buccal cell samples were collected from students in the classroom by trained graduate students using cotton buccal brushes. The research team was trained in ethical research conduct and was attentive to the cultural norms and values of the participating communities. Classroom teachers were instructed to notify participants to refrain from eating, smoking, drinking alcohol, or chewing gum for 30 min prior to sample collection. The entire sampling process took approximately 30 min to complete. Prior large-scale genetic studies have demonstrated that Han Chinese populations, particularly those from the same geographic area, exhibit a low degree of genetic substructure (Chen et al., 2009; Xu et al., 2009). In such genetically homogeneous samples, the risk of population stratification biasing genetic association results is considered minimal.

### 2.3. Measures

#### 2.3.1. Parenting Stress

Parenting stress was measured using the PSI-SF (Parenting Stress Index-Short Form; Abidin, 1983). Its Chinese adaptation has been validated and shown to have strong psychometric reliability (Luo et al., 2019). This scale includes 15 items, each rated on a 5-point Likert scale (1 = strongly disagree, 5 = strongly agree), and assesses three dimensions: parental distress (5 items, e.g., “I can’t cope with novel things”) and dysfunctional parent–child interaction (5 items, e.g., “My child rarely does anything that makes me feel satisfied”)and difficult child (5 items, e.g., “I feel my child is very emotional and easily distressed.”). A composite score was calculated by averaging item responses, with higher scores indicating greater parenting stress severity. The PSI-SF showed high internal consistency in this study with a Cronbach’s α of 0.95.

#### 2.3.2. Maladaptive Parenting

Parental harsh punishment and Psychological control were measured using the parent-reported harsh punishment and psychological control subscales of the parent-version Parenting Behavior Scale (Janssens et al., 2015). The harsh punishment subscale comprises 4 items (e.g., “I spank my child when he/she is disobedient or naughty”), while the psychological control subscale includes 8 items (e.g., “I am less friendly with my child if he/she doesn’t see things my way”). Responses were rated on a 5-point scale from 0 (never) to 4 (often). The mean score of harsh punishment and psychological control subscales was calculated to represent parental maladaptive parenting practices, with higher scores indicating greater severity. The parent-version scale has shown good reliability and validity among Chinese student (Luo et al., 2019), and in the present study, Cronbach’s α coefficients for the harsh punishment and psychological control subscales were 0.82 and 0.87, respectively.

#### 2.3.3. Social Anxiety

Social anxiety was measured using the SASC (Social Anxiety Scale for Children; La Greca et al., 1988). Its Chinese adaptation has been validated and shown to have strong psychometric reliability (Li et al., 2006). This self-report tool includes 10 items, each rated on a 3-point Likert scale (0 = not at all, 2 = all the time), and assesses two dimensions: Fear of Negative Evaluation (6 items, e.g., “I worry about being teased”) and Social Avoidance and Distress (4 items, e.g., “I get nervous when I talk to new kids”). A composite score was calculated by averaging item responses, with higher scores indicating greater social anxiety severity. The SASC showed high internal consistency in this study with a Cronbach’s α of 0.92.

#### 2.3.4. Genotyping

DNA (deoxyribonucleic acid) was extracted from buccal cell samples. The SNaPshot assay was employed to detect the polymorphisms of rs4713916, rs1360780, rs3800373, and rs9296158 via fluorescence capillary electrophoresis on an ABI 3730xl instrument (CE platform/next-generation sequencing platform). Genotyping results were interpreted and analyzed using the GeneMaker v3.0 software system. For quality assurance, 5.0% of samples were randomly selected for repeated genotyping, yielding a concordance rate exceeding 99.0%. All four SNPs achieved a 100% genotype call rate in the final analytic sample (N = 1774). All MAFs exceeded 5%. Kang et al. (2019) examined multiple FKBP5 SNPs in an East Asian sample and reported low pairwise LD among these variants. The findings suggest that the four selected SNPs capture relatively independent genetic signals within FKBP5. The genotypes of the four FKBP5 gene SNPs (Single nucleotide polymorphism) are summarized in Table 1. The rs1360780 genotype distribution showed a statistically significant deviation from Hardy–Weinberg equilibrium (χ^2^ = 5.56, *p* = 0.02). Prior research has demonstrated that cumulative genetic scores are relatively robust to minor deviations at individual loci (e.g., Starr & Huang, 2019; Zhou & Gong, 2023).

In accordance with previous research (e.g., Zhou & Gong, 2023), we coded the FKBP5 gene based on the count of minor alleles across the four SNPs: rs4713916 (AA = 2, GA = 1, GG = 0); rs1360780 (TT = 2, CT = 1, CC = 0); rs3800373 (CC = 2, CA = 1, AA = 0); and rs9296158 (AA = 2, GA = 1, GG = 0). Then, grounded in the multilocus genetic profile score (MGPS) framework (Bogdan et al., 2013), a cumulative genetic score was calculated by summing these allele counts. A higher score indicates a greater number of minor alleles across the four loci. The FKBP5-related cumulative genetic score ranged from 0 to 8, with the following score distribution frequencies: 0 (N = 765, 43.1%); 1 (N = 183, 10.3%); 2 (N = 30, 1.7%); 3 (N = 119, 6.7%); 4 (N = 498, 28.1%); 5 (N = 69, 3.9%); 6 (N = 13, 0.7%); 7 (N = 32, 1.8%); and 8 (N = 65, 3.7%).

#### 2.3.5. Demographic Variables

Demographic variables were used as controls. Students reported age, grade, and gender (0 = boy, 1 = girl). Parents provided data on their educational attainment (1 = never been to school to 8 = doctorate), occupational stability (1 = stable, 2 = unstable), and the student’s physical health status (1 = very poor to 5 = very healthy) via questionnaires.

### 2.4. Data Analysis

First, the intraclass correlation coefficients (ICCs) for our primary study variables were calculated. The ICCs for both child social anxiety and parenting stress were less than 0.05, indicating that class-level clustering accounted for negligible variance in our key measures. Second, descriptive statistics and bivariate correlations for the main study variables were analyzed using the statistical software Mplus version 8.0. Third, after controlling for covariates, the direct effect of parental parenting stress on children’s social anxiety was examined. Fourth, the indirect effect of maladaptive parenting in the relationship between parental parenting stress and children’s social anxiety was examined, again after controlling for covariates. Fifth, a model was conducted to examine the moderating effects of FKBP5 gene variation. The interaction between the FKBP5-related cumulative genetic score and parental parenting stress/maladaptive parenting was created, and data for parental parenting stress/maladaptive parenting were mean-centered prior to analysis to reduce multicollinearity between product terms. After detecting an interaction effect, simple slope analysis was carried out.

The percentile bootstrapping approach (N = 5000) was used to assess the significance and strength of the indirect effect. When the confidence intervals did not include zero, the indirect effect was considered statistically significant. Model fit was evaluated using the CFI (Comparative Fit Index) and the RMSEA (root-mean-square error of approximation). CFI and TLI (Tucker–Lewis Index) values above 0.90, along with RMSEA values of 0.06 or lower, are generally regarded as indicators of a good fit between the hypothesized model and the observed data (Marsh et al., 2004).

## 3. Results

### 3.1. Descriptive Statistics

Table 2 shows the means and standard deviations of the study variables, as well as the correlations between all study variables. Specifically, parental parenting stress is positively associated with children’s social anxiety (r = 0.27, *p* < 0.01) and maladaptive parenting (r = 0.57, *p* < 0.01). Maladaptive parenting is positively associated with children’s social anxiety (r = 0.20, *p* < 0.01). The FKBP5 related cumulative genetic score is unrelated to parental parenting stress, maladaptive parenting and children’s social anxiety.

### 3.2. The Effect of Parenting Stress on Children’s Social Anxiety

First, the model is constructed in order to examine the effect of parental parenting stress on children’s social anxiety. The model showed an accepted fit to the data, RMSEA = 0.00, CFI = 1.00, TLI = 1.00. The results show that the effect of parental parenting stress on children’s social anxiety is statistically significant (*β* = 0.25, *p* < 0.001), after controlling for the covariates (i.e., gender, grade, maternal/paternal education levels, maternal/paternal occupational stability and students’ physical status).

### 3.3. The Indirect Effect of Maladaptive Parenting

**Figure 2:**
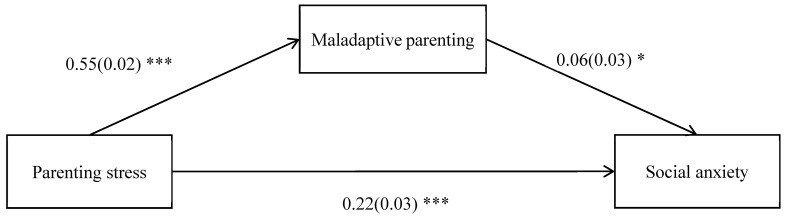
The indirect effect of maladaptive parenting between parental parenting stress and children’s social anxiety. The values outside the parentheses are standardized regression coefficients and the values inside the parentheses are standard errors. The covariates were all controlled on maladaptive parenting and children’s social anxiety. *** *p* < 0.001. * *p* < 0.05.

Then, the model is constructed in order to examine the indirect effect of maladaptive parenting between parental parenting stress and children’s social anxiety. The model showed an accepted fit to the data, RMSEA = 0.00, CFI = 1.00, TLI = 1.00. As is shown in Figure 2, after controlling for the covariates on maladaptive parenting and children’s social anxiety, the effect of parental parenting stress on maladaptive parenting (*β* = 0.55, *SE* = 0.02, *p* < 0.001) is statistically significant. In turn, the effect of maladaptive parenting on children’s social anxiety (*β* = 0.06, *SE* = 0.03, *p* < 0.05) is statistically significant. The direct effect of parental parenting stress on children’s social anxiety is statistically significant (*β* = 0.22, *SE* = 0.03, *p* < 0.001). Maladaptive parenting thus plays a partly indirect effect in the relation between parental parenting stress and children’s social anxiety. Bootstrapping employing 5000 samples shows that the confidence intervals of the indirect effect of social anxiety do not include zero (*β* = 0.04, *SE* = 0.02, 95% *CI* = [0.00, 0.07]), indicating statistical significance.

### 3.4. The Moderating Role of FKBP5 Related Cumulative Genetic Score

**Figure 3:**
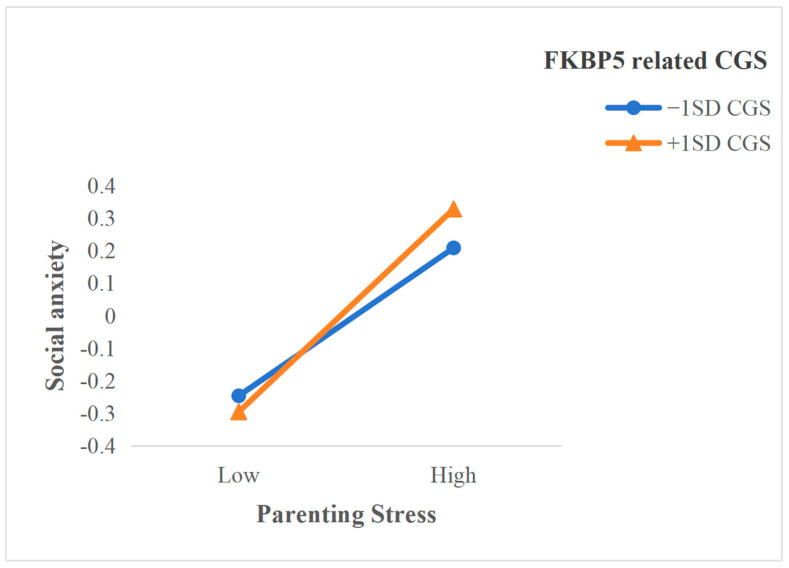
Interactive effect of Parental parenting stress and FKBP5 related cumulative genetic score on children’s social anxiety.

Finally, the model is constructed by grouping the FKBP5 cumulative genetic score (CGS) into three categories: low (CGS = 0, N = 765), moderate (CGS = 1–4, N = 830), and high (CGS = 5–8, N = 179) in order to examine the moderating role of FKBP5 related cumulative genetic score. This grouping avoids extremely small cells while preserving the conceptual gradient of genetic risk. The model demonstrates an accepted fit for the data (RMSEA = 0.004, CFI = 1.00, TLI = 0.99). The results (see Table 3) shows that the interaction effect of parental parenting stress and the cumulative genetic score on children’s social anxiety is marginally significant, but the interaction effect of maladaptive parenting and the cumulative genetic score on children’s social anxiety is insignificant. This result indicates that the FKBP5-related cumulative genetic scores potentially moderate the effect of parental parenting stress on children’s social anxiety. A simple slope test shows that parenting stress was more strongly associated with child social anxiety among children with higher genetic risk (see Figure 3).

## 4. Discussion

The present study, drawing on a sample of 1774 Chinese fourth- to sixth-grade students and their parents, examined the relationships among parental parenting stress, maladaptive parenting (specifically harsh punishment and psychological control), FKBP5 gene variation, and children’s social anxiety. The results revealed that parental parenting stress significantly and positively predicted children’s social anxiety; maladaptive parenting partially mediated this relationship; and FKBP5 gene variation moderated the direct effect of parental parenting stress on children’s social anxiety. These findings offer new insights into how gene–family environment interactions jointly influence the development of social anxiety in children.

### 4.1. The Direct Effect of Parenting Stress on Children’s Social Anxiety

First, the present study found that parental parenting stress was significantly and positively associated with children’s social anxiety, and this effect remained robust after controlling for demographic covariates (i.e., gender, grade, maternal/paternal education levels, maternal/paternal occupational stability, and students’ physical health status). This result is consistent with prior research. Rorem (2020) found that dysfunctional parent–child interactions at age 3 were significantly associated with children’s internalizing behaviors (including anxiety and depression) at age 5. Jia et al. (2024) demonstrated that maternal parenting stress was directly associated with children’s socioemotional problems. Compared with previous studies that have primarily focused on preschool-aged children, the present study, with a sample of fourth- to sixth-grade students (aged 10–14 years), suggests that even during middle childhood, parental parenting stress remains a relevant correlate of children’s social anxiety. Despite the fact that children in this age group increasingly engage in peer comparison and complex social interactions, the family emotional climate continues to show an association with their mental health. However, due to the cross-sectional design, causal direction cannot be inferred.

### 4.2. The Partial Mediating Role of Maladaptive Parenting

Second, the present study found that parental parenting stress was significantly associated with maladaptive parenting, and that maladaptive parenting, in turn, was significantly associated with children’s social anxiety, yielding a significant indirect effect. This result is largely consistent with the coercive family process model (Moed, 2024), which posits that high parenting stress escalates into harsh parenting practices, thereby increasing children’s internalizing problems. However, compared with prior research, the indirect effect observed in this study was relatively small.

One possible interpretation, albeit speculative, relates to the Chinese cultural context. In traditional Chinese beliefs, adages such as “hitting is caring, scolding is loving” and “a dutiful son is raised under the rod” reflect a degree of normalization and even moralization of harsh discipline (Lian et al., 2024). Within such a cultural climate, parental corporal punishment or psychological control may not be perceived as “maladaptive”, but rather construed as an educational practice intended for the child’s own good. Consequently, parents may exhibit social desirability bias when reporting such behaviors, underestimating the actual frequency and intensity of maladaptive and thereby attenuating its observed association with children’s social anxiety. Furthermore, cultural norms may also shape children’s cognitive appraisal of maladaptive parenting behaviors. In family environments where harsh discipline is regarded as normative, children may interpret parental scolding and hitting as expressions of “concern” rather than “rejection”, which could diminish the adverse impact on anxiety (Cheah et al., 2019). However, this interpretation remains speculative, as the present study did not directly measure cultural acceptance of harsh discipline nor children’s appraisals. Moreover, noting these cultural beliefs does not imply that harsh discipline is harmless; rather, it highlights the need for culturally sensitive research that directly examines these mechanisms.

Moreover, it is noteworthy that even after incorporating the mediator, the direct association between parenting stress and children’s social anxiety remained significant. This finding suggests that parenting stress may be directly perceived by children, in addition to being associated with child outcomes via parenting behaviors. Future cross-cultural research is needed to further elucidate the mechanisms linking parenting stress to children’s social anxiety and to inform interventions aimed at mitigating its potential negative impact.

### 4.3. The Moderating Role of the FKBP5-Related Cumulative Genetic Score

Finally, the present study found that FKBP5 gene variation showed a marginally significant moderating effect on the relationship between parental parenting stress and children’s social anxiety. Simple slope test (see Figure 3) suggested parenting stress was more strongly associated with child social anxiety among children with higher genetic risk. In other words, even when parents experiencing high levels of parenting stress do not exhibit overt maladaptive behaviors, children with higher genetic risk appeared to be more sensitive to parenting stress. Given the marginal significance, this finding should be interpreted as preliminary and requires replication in independent samples.

This pattern is theoretically consistent with the diathesis–stress model (Grillo, 2025). It is plausible that FKBP5 risk alleles impair the negative feedback function of glucocorticoid receptors, which may contribute to chronically elevated cortisol levels and compromised prefrontal cortex function (Zannas & Binder, 2014; Zhou & Gong, 2023). However, these neurobiological mechanisms were not directly measured in the present study, and thus this interpretation remains speculative and should be tested in future research.

Furthermore, supplementary analyses (see Appendix A) revealed that the SNPs rs4713916 and rs1360780 each moderated the relationship between parental parenting stress and children’s social anxiety. This may indicate that, while the cumulative genetic score serves as a broad indicator of environmental sensitivity, these two SNPs may carry disproportionate weight. However, given the exploratory nature of these supplementary analyses and the marginal significance of the primary interaction, these results should be interpreted with caution.

Unexpectedly, the interaction between FKBP5 gene variation and maladaptive behaviors was not significant. One possible explanation is that HPA-axis-related genetic variants may be more sensitive to chronic, pervasive daily stress (parenting stress) than to more acute stressors (maladaptive behaviors) (Zeng et al., 2024). Maladaptive behaviors, when they occur, may represent relatively intense stressors that could push HPA-axis activation toward a ceiling effect, thereby attenuating the incremental contribution of genetic variation. Alternatively, as discussed in Section 4.2, within the Chinese cultural context, traditional beliefs such as “hitting is caring, scolding is loving” may normalize harsh discipline (Wang et al., 2018). Parents may tend to underreport maladaptive behaviors, and children may reinterpret such behaviors as expressions of “concern”, thereby reducing the perceived threat level of maltreatment as a stressor (Huang & Frost, 2025). These explanations remain speculative, as the present study did not directly measure cultural beliefs or children‘s appraisals. Future research should incorporate a broader array of genetic variants and employ multiple methods (e.g., observational measures) to assess parenting behaviors, and directly examine cultural and cognitive factors to further investigate this issue.

### 4.4. Theoretical Integration

Taken together, the present findings are consistent with a model in which parenting stress is associated with children’s social anxiety both directly and indirectly through maladaptive parenting. Cumulative genetic risk (operationalized in this study as the FKBP5-related polygenic score) showed a marginally significant moderating effect on the direct path, suggesting a possible role as a moderator of environmental sensitivity. However, given the cross-sectional design and the marginal significance of the genetic interaction, these findings should be interpreted as preliminary and require replication.

This model offers several potential theoretical insights for understanding of children’s socioemotional development. First, the mediating role of maladaptive parenting in the relationship between parenting stress and children’s social anxiety is consistent with the idea that, within high-stress parenting families, maladaptive parenting toward children serves as one pathway through which parenting stress is linked to child outcomes. Second, the marginally significant G × E interactions is compatible with the hypothesis that children with higher FKBP5 cumulative genetic risk scores are particularly sensitive to parental parenting stress, which in turn heightens their vulnerability to social anxiety. Nevertheless, these interpretations remain tentative pending replication in longitudinal and independent samples.

### 4.5. Practical Implications

The findings of the present study, while preliminary, suggest several potential directions for prevention and intervention. For children carrying a higher number of FKBP5 risk alleles, interventions might benefit from prioritizing the reduction in parents’ daily parenting stress. Specific strategies that could be considered include mindfulness-based parenting programs (e.g., Mindfulness-Based Stress Reduction adapted for parents), parent stress management workshops, and enhancing social support networks through school or community resources. Even non-maltreatment levels of parenting stress may be associated with elevated social anxiety in this potentially vulnerable group, although this interpretation awaits replication.

Concurrently, the prevention of maladaptive parenting behaviors (e.g., through positive parenting training such as Parent–Child Interaction Therapy or similar evidence-based programs) remains important. Such interventions may be particularly for children at high genetic risk who have already been exposed to harsh or psychologically controlling parenting. In contexts of limited resources, universal parenting stress interventions may yield broader population benefits, while more intensive resources could be considered for families characterized by both high parenting stress and high genetic risk.

It is important to note that these recommendations are speculative and derived from a single cross-sectional study with a marginally significant genetic interaction. Genetically informed stratified interventions should be viewed as a direction for future hypothesis-driven research rather than as directly supported by the current findings. Any practical applications would require replication in longitudinal studies, independent samples, and, ultimately, randomized controlled trials.

### 4.6. Limitations and Future Directions

The present study has several limitations. First, the cross-sectional design precludes causal inference. Although the mediation model was theoretically driven, the model were saturated; thus, model fit for these models could not be independently assessed. Future research should employ multi-wave longitudinal designs to establish the temporal ordering among parenting stress, maladaptive behaviors, and social anxiety. Second, common method bias may be present (parents reported both parenting stress and maladaptive behaviors). However, interaction effects (moderation) are less susceptible to common method bias (Siemsen et al., 2010). Future studies could incorporate child-reported parenting behaviors or observational measures to mitigate this bias. Third, this study focused exclusively on FKBP5-related loci. Although FKBP5 occupies a central role in HPA-axis regulation, other systems (e.g., serotonergic and dopaminergic pathways) may also contribute to cumulative genetic risk; integrating polygenic scores across multiple systems could yield greater predictive power. Fourth, the sample was drawn from a specific region in China, and caution is warranted in generalizing the findings to other cultural or ethnic populations. Finally, this study did not measure variables such as school support that may moderate genetic effects; future research should explore these potential moderators further.

## 5. Conclusions

In conclusion, the present study found that parenting stress is associated with children‘s social anxiety both directly and indirectly through maladaptive parenting. Furthermore, the FKBP5 cumulative genetic risk showed a marginally significant moderating effect on the direct path, suggesting a possible role in environmental sensitivity. However, given the cross-sectional design and the marginal significance of the genetic interaction, these findings should be interpreted as preliminary and require replication in longitudinal and independent samples. The findings suggest that daily parenting stress may be a target in gene–environment research, particularly within non-clinical populations. By adopting a polygenic cumulative score approach within a moderated mediation model, this study provides a basis for future research examining how genetic factors may be associated with children‘s social anxiety. Genetically informed interventions remain speculative at this stage and would require substantial further evidence before any practical application could be considered.

## Figures and Tables

**Table 1:** The genotypes of the four SNPs of the FKBP5 gene.

Genotypes				Hardy−Weinberg Equilibrium		
				χ^2^	df	*p*
rs4713916	GG (N = 1051)	GA (N = 641)	AA (N = 82)	1.59	1	0.21
rs1360780	CC (N = 961)	CT (N = 715)	TT (N = 98)	5.56	1	0.02
rs3800373	AA (N= 984)	CA (N = 680)	CC (N = 110)	0.27	1	0.60
rs9296158	GG (N = 814)	AG (N = 777)	AA (N = 183)	0.01	1	0.90

**Table 2:** Descriptive statistics and correlations for the study variables.

	M	SD	1	2	3	4	5	6	7	8	9	10	11
1. Gender	—	—	—										
2. Grade	—	—	0.03	—									
3. F-edu	3.33	0.78	−0.04	−0.01	—								
4. M-edu	3.15	0.82	−0.05 *	0.05 *	0.51 **	—							
5. F-ocup	1.36	0.48	0.03	0.01	−0.19 **	−0.17 **	—						
6. M-ocup	1.60	0.49	−0.01	0.04	−0.19 **	−0.24 **	0.45 **	—					
7. Body status	4.27	0.79	−0.01	−0.01	0.01	−0.01	−0.09 **	−0.06 *	—				
8. Parenting stress	2.27	0.73	−0.01	−0.05 *	−0.03	−0.08 **	0.15 **	0.12 **	−0.22 **	—			
9. Mal-parenting	2.01	0.56	0.01	−0.01	0.00	−0.04	0.14 **	0.12 **	−0.17 **	0.57 **	—		
10. Social anxiety	1.42	0.44	−0.08 **	−0.05 *	0.01	−0.002	0.04	0.05	−0.13 **	0.27 **	0.20 **	—	
11. FKBP5-CGS	2.12	2.29	0.04	0.03	−0.02	0.002	0.02	0.02	−0.07 **	0.02	0.04	0.02	—

Note. F-edu = Paternal education levels; M-edu = Maternal education levels; F-ocup = Paternal occupational stability; M-ocup = Maternal occupational stability; Mal-parenting = maladaptive parenting; CGS = cumulative genetic score. ** *p* < 0.01. * *p* < 0.05.

**Table 3:** Results of the moderated mediation model.

	Mal-Parenting		Social Anxiety	
	*β*	*SE*	*β*	*SE*
Gender	0.01	0.02	−0.08 ***	0.02
Grade	0.01	0.02	−0.03	0.02
F-edu	0.04	0.02	0.002	0.03
M-edu	0.001	0.03	0.01	0.03
F-ocup	0.05 *	0.02	−0.01	0.03
M-ocup	0.03	0.02	0.02	0.03
Body status	−0.05 *	0.04	−0.07 *	0.03
Parenting stress	0.55 ***	0.02	0.18 ***	0.04
Mal-parenting	—	—	0.06 *	0.03
FKBP5 related CGS	—	—	0.02	0.02
Parenting stress × FKBP5 CGS	—	—	0.05 †	0.03
Mal-parenting × FKBP5 CGS	—	—	0.01	0.03

Note. F-edu = Paternal education levels; M-edu = Maternal education levels; F-ocup = Paternal occupational stability; M-ocup = Maternal occupational stability; Mal-parenting = maladaptive parenting; CGS = cumulative genetic score. *** *p* < 0.001. * *p* < 0.05. † *p* < 0.1.

**Figure A1:**
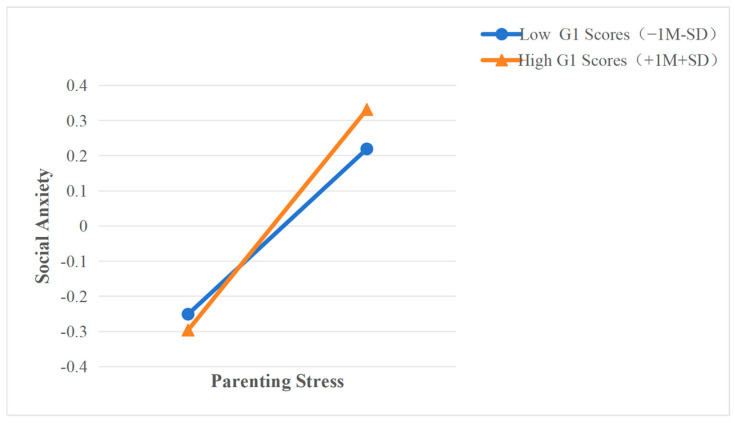
Interactive effect of parenting stress and G1 scores on children’s social anxiety.

**Figure A2:**
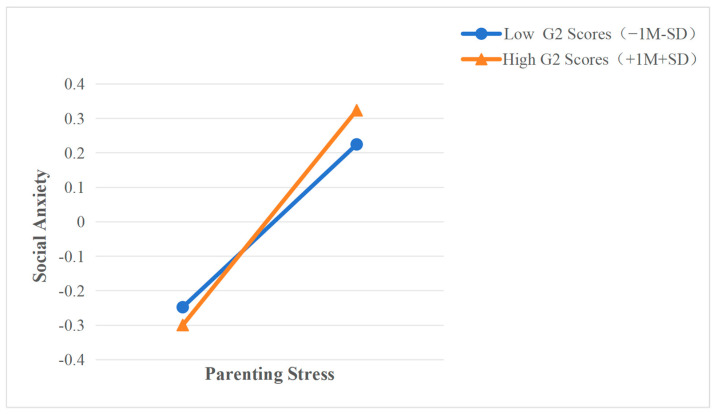
Interactive effect of parenting stress and G2 scores on children’s social anxiety.

## Data Availability

The original contributions presented in this study are included in the article. Further inquiries can be directed to the corresponding author.

## Abbreviations

The following abbreviations are used in this manuscript:

HPA	Hypothalamic–pituitary–adrenal
FKBP5	FK506 binding protein 5
SNP	Single nucleotide polymorphism
PSI-SF	Parenting Stress Index–Short Form
SASC	Social Anxiety Scale for Children
MBSR	Mindfulness-based stress reduction
G × E	Gene–environment interaction
CFI	Comparative Fit Index
RMSEA	Root-mean-square error of approximation
TLI	Tucker–Lewis Index
ADHD	Attention-deficit/hyperactivity disorder
DNA	Deoxyribonucleic acid
CGS	Cumulative genetic score

## Appendix A

We also examine the moderating role of the four SNPs (G1:rs4713916, G2: rs1360780, G3: rs3800373, G4: rs9296158) between parental parenting stress and children’s social anxiety, respectively. These analyses are exploratory and should be interpreted with caution.

### Appendix A.1. The Moderating Role of G1 (rs4713916)

The results show that the interaction effect of parental parenting stress and G1 (rs4713916) on children’s social anxiety is statistically significant (β (SE) = 0.06 (0.03), *p* < 0.05). A simple slope test shows that parenting stress was more strongly associated with child social anxiety among children with higher genetic scores (Figure A1). Specifically, the slopes for genetic scores of 0 to 2 are all significant, ranging from b (SE) = 0.18 (0.04), *p* < 0.001 for an index value of 0 to b (SE) = 0.34 (0.07), *p* < 0.001 for an index value of 2.

### Appendix A.2. The Moderating Role of G2 (rs1360780)

The results show that the interaction effect of parental parenting stress and G2 (rs1360780) on children’s social anxiety is marginally significant (β (SE) = 0.05 (0.03), *p* < 0.1). A simple slope test shows that parenting stress was more strongly associated with child social anxiety among children with higher genetic scores (Figure A2). Specifically, the slopes for genetic scores of 0 to 2 are all significant, ranging from b (SE) = 0.18 (0.04), *p* < 0.001 for an index value of 0 to b (SE) = 0.32 (0.06), *p* < 0.001 for an index value of 2.

### Appendix A.3. The Moderating Role of G3 (rs3800373)

The results show that the interaction effect of parental parenting stress and G3 (rs3800373) on children’s social anxiety is insignificant (β (SE) = 0.04 (0.03), *p* = 0.14).

### Appendix A.4. The Moderating Role of G4(rs9296158)

The results show that the interaction effect of parental parenting stress and G4 (rs9296158) on children’s social anxiety is insignificant (β (SE) = 0.03 (0.03), *p* = 0.34).
